# Three types of bidirectional leader development in triggered lightning flashes

**DOI:** 10.1038/s41598-023-32003-x

**Published:** 2023-03-31

**Authors:** Rui Su, Fukun Wang, Jianguo Wang, Li Cai, Jinxin Cao, Junlin Wang

**Affiliations:** Engineering Research Center of Ministry of Education for Lightning Protection and Grounding Technology, School of Electrical Engineering and Automation, Wuhan University, Wuhan, 430072 China

**Keywords:** Space physics, Engineering, Physics

## Abstract

Eight cases of bidirectional leader (BL) development in artificially triggered lightning flashes are reported with synchronous high-speed camera images and electric field signals. Based on optical progressing characteristics, the eight cases can be divided into three types: a reflection type, a discontinuity type, and an inducement type. For the reflection type, the tail of a dart leader may begin to extend backward when the leader’s head reaches a branch point, or the top of the exploded triggering wire. For the discontinuity type, the initiation of a bidirectional leader below a decayed attempted leader may occur more than once preceding one return stroke. For the inducement type, the approach of another leader with the same polarity will turn a dart leader into a bidirectional leader. The reflection type and inducement type are first observed here. Two cases of the discontinuity type are observed, and both are multiple-bidirectional leaders observed for the first time. For the reflection type and inducement type, there are fluctuations in the electric field related to the BL development. The dissipation of the downward leader slows down the negative increase of the electric field. Once the BL development starts, the downward negative end of the BL moves towards the ground with the E-field negatively increasing. For the discontinuity type, the close electric field result shows no fluctuations. The BL development has a much longer duration than the other two BL types.

## Introduction

The concept of bidirectional leader (BL) propagation that a leader has two ends developing simultaneously in opposite directions with opposite polarity was first proposed by Kasemir et al.^1^. The dynamics of the two leader ends in terms of the propagation speed can be different^2,3^. The positive end can propagate faster^4,5^ or slower than the negative end^6,7^, or the two ends can propagate at similar speeds^8^.

The BLs, which have been documented by various observations, can be divided into two main kinds. The first kind of BL originates from metallic objects such as aircraft^6^, suspended conductors^9^, and floating triggering wires^10,11^. The second kind of BL is electrodeless discharge, whose formation has been concluded by Tran and Rakov^2^ to have four main scenarios: (1) initiation of lightning in the cloud^12^, (2) lightning channel branching process^13,14^, (3) space leader involved in the negative leader step-formation process^15^, and (4) recoil-leader-type process giving rise to K-changes, dart leaders, and M-components^16^.

Recoil leaders, which usually develop in a bidirectional way, are fast, short-lasting leaders retracting the decaying channel of a preceding leader in a retrograde fashion^3,17^. Recoil leaders were initially concluded to be negative leaders retracting the decaying channel of a positive leader^18,19^. Recent observations have revealed that recoil leaders can also be formed in decaying negative leader channels. Jiang et al.^20^ reported the high-speed video evidence of a dart leader with bidirectional development in the decaying negative leader channel of an upward cloud-to-ground lightning flash. Qie et al.^4^ reported two cases of BL development in two negative artificially-triggered lightning flashes. The three cases of BL development observed by Jiang et al.^20^ and Qie et al.^4^ shared some similar characteristics. All these BLs originated from a point below the termination point of a preceding attempted leader that just died out. The positive ends of these BLs retracted the decaying channel of the preceding attempted leader at higher speeds than the negative ends which propagated downwards along the remnants of the channel created by the previous stroke or initiate a continuous current. Jiang et al.^21^ reported an intracloud lightning flash where a positive leader from the cloud induced a bidirectional luminous segment ahead of the leader tip. It follows that various reasons contribute to the formation of bidirectional leaders. Thus, more observation cases are needed to analyze and study, which is very important to understand the lightning discharge process.

Here we exhibit eight cases of bidirectional leader development in a residual channel of several artificially triggered lightning flashes. Synchronous high-speed camera (HSC) images, current, and electric field signals are processed and reported to show different progressing characteristics of bidirectional leaders. In addition, the eight cases are divided into three types. For each case, bidirectional leaders' initiation and development process are introduced, along with an analysis of electric field waveforms. This paper’s work will help deepen the understanding of bidirectional leaders by showing various types of bidirectional leaders.

## Results

The eight cases were contained in six negative triggered lightning flashes, of which one was the altitude-triggered lightning flash and the other five were classical triggered lightning flashes. Except for the triggering type of each flash, Table 1 lists the numbers of return strokes (RSs), the peak value of the studied RSs current, the observation distances of recorded current and electric field signals, and the corresponding relationship between flashes and cases. No relevant current and electric field data were recorded in Flash F. The HSC did not capture the whole process of Flash F, only including four return strokes.

**Table 1:** The basic information about six triggered lightning flashes.

Flashes	Classical/altitude	RSs	Current (kA)	Electric field	Cases	The studied RS
A	Classical	12	8.17	80 m	1	8th
B	Classical	7	9.05	18 m 130 m	2	4th
C	Classical	13	5.69	58 m 80 m 1550 m	3	4th
D	Altitude	8	–	130 m 1550 m	4	3rd
					5	4th
					8	8th
E	Classical	9	13.13	130 m	6	4th
F	Classical	4	–	–	7	4th

### Optical features of BL development

**Figure 1:**
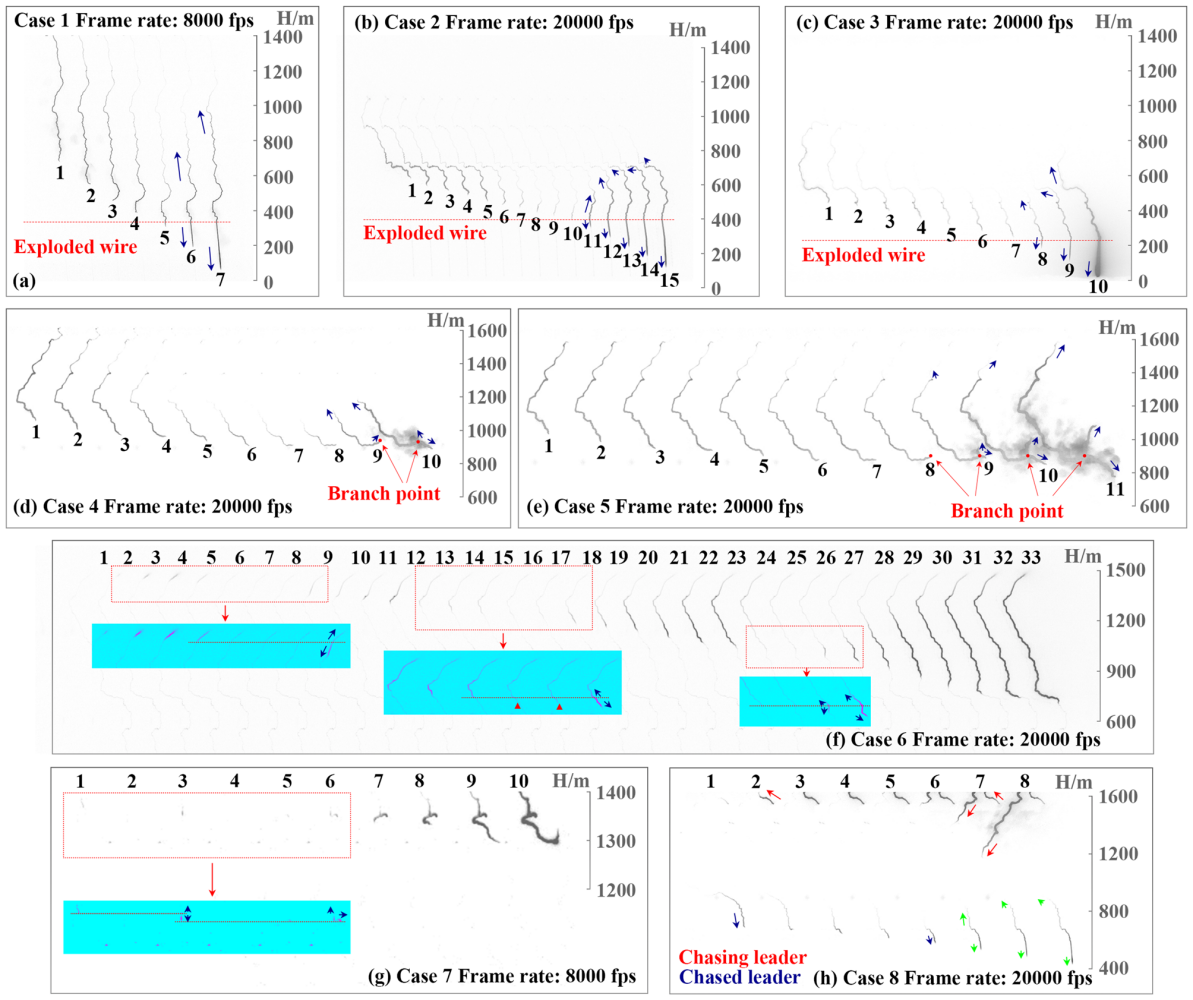
Consecutive frames revealing the eight cases of BL developments with background luminosity removed, contrast-enhanced, and intensity inverted. Some areas are recolored for a better presentation. Blue and green arrows show the bidirectional propagation of leaders.

Figure 1 shows the arranged consecutive frames revealing the eight cases of BL development. Case 1 contained a BL preceding the 8th return stroke (RS) of Flash A, which was triggered at 08:22:36 (UTC), 5 June 2019, as shown in Fig. 1a. In Frames 1 through 5, the dart or dart-stepped leader (only referred to as dart leader below) propagated down near the top of the exploded triggering wire, while the length of the luminous channel was shorter and shorter. Then in Frames 6 and 7, the dart leader turned to be a BL without any visible pauses, indicated by the backward propagating leader tail. In the next frame of Frame 7, the BL induced the 8th RS. From Frames 5 to 7, the positive end of the BL moved upward with an average speed of 2.09 × 10^6^ m/s, while the negative end extended downward with an average speed of 9.71 × 10^5^ m/s.

Case 2 contained a BL before the 4th RS of Flash B triggered at 09:15:50 (UTC), 30 June 2019, as shown in Fig. 1b. The dart leader had a decaying downward propagation in Frames 1 through 10. In Frame 10, the leader’s head was near the top of the exploded triggering wire. Then in Frame 11, the leader began to propagate bidirectionally. The 4th RS was induced by the BL in the next frame of Frame 15. From Frames 10 to 15, the positive end of the BL moved upward with an average speed of 1.97 × 10^6^ m/s, while the negative end extended downward with an average speed of 1.30 × 10^6^ m/s.

Case 3 recorded a BL before the 4th RS of Flash C which was triggered at 07:15:04 (UTC), 2 July 2019, as illustrated in Fig. 1c. The initial decaying downward propagation can also be found in Case 3 from Frame 1 to Frame 7. In Frame 8, the leader began to propagate in the channel of the exploded triggering wire, while the tail of the leader started extending upwards. From Frames 8 to 10, the positive end of the BL moved upward with an average speed of 3.90 × 10^6^ m/s, while the negative end extended downward with an average speed of 1.92 × 10^6^ m/s.

Figure 1d,e depict Case 4 and Case 5, where two BLs induced the 3rd and 4th RS of Flash D, respectively. Flash D, an altitude-triggered lightning flash, was triggered at 10:02:22 (UTC), 7 July 2019. The two leaders were also decaying dart leaders propagating unidirectionally before they reached the branch points. The tail of the two leaders turned to extend backward when the upward propagating branches were produced at the two branch points, indicating that the two leaders had turned to be BLs. From Frames 8 to 10 in Case 4, the upward positive end of the BL moved with an average speed of 2.14 × 10^6^ m/s. Between Frames 8 and 9, the speed of the downward negative end was 1.11 × 10^6^ m/s. Then the downward negative end branched to two channels in Frame 10, having two propagation speed of 1.19 × 10^6^ and 1.79 × 10^6^ m/s. In Case 5, the upward positive end of the BL had an average speed of 2.66 × 10^6^ m/s from Frames 9 to 11. The downward negative end branched to two ends, extending with an average speed of 1.71 × 10^6^ and 1.39 × 10^6^ m/s.

Figure 1f shows Case 6 mentioned in^22^, where a multiple-bidirectional leader initiated the 4th RS of Flash E triggered at 09:12:47 (UTC), 30 June 2019. This leader is a multiple-bidirectional one whose propagation involves such a process that a bidirectional leader is initiated, or a unidirectional leader turns to be a bidirectional leader, more than once. The leader entered the field of view in Frame 1 and extended downwards in the next three frames. Then the leader channel became invisible even if in the recolored and contrast-enhanced portion of Frames 5, 6, and 7. The tail of the leader did not appear in Frames 1 through 7 therefore it is hard to figure out whether the leader was a bidirectional one. In Frame 8, a BL was initiated, which will be referred to as the first BL below. If the first BL originated from the height where the preceding leader’s head stopped in Frame 4, the negative end of the first BL was more luminous than the positive end in Frame 8. From Frame 8 to 14, the luminosity of the channel was mainly concentrated in the leader’s head. In Frames 15 and 16, the leader channel became invisible again, but the leader channel was found to have quite low luminosity and kept extending downward in the recolored and contrast-enhanced portion. The positions of the leader’s head were indicated by the red triangles. From Frames 8 to 15, the positive end of the BL moved upward with an average speed of 6.01 × 10^5^ m/s, while the negative end extended downward with an average speed of 4.80 × 10^5^ m/s. If the first BL was considered to have been terminated in Frame 15 for its quite low luminosity, then the BL that originated in Frame 17 is the second BL. When the second BL originated, it propagated in a bidirectional way as shown in Frames 17 through 19. During this period, the average speed of the upward positive end and the downward negative end were 8.20 × 10^5^ and 4.73 × 10^5^ m/s respectively. The tail of the second BL once decayed in Frame 20 and then extended upwards again in Frame 21, indicating that the second BL propagated in a bidirectional-unidirectional-bidirectional way. The head of the second BL began to decay in Frame 24, and the third BL originated in Frame 26. From Frames 26 to 33, the positive end of the third BL rose about 600 m in height with an average speed of 1.70 × 10^6^ m/s, while the negative end dropped less than 400 m within 0.4 ms with an average speed of 9.58 × 10^5^ m/s. The downward propagation of the third BL eventually reached the ground and induced the return stroke. It should be noted that the speed of the upward positive end of three BLs was calculated only when the upward positive end was not beyond the top view of the HSC.

Figure 1g shows Case 7 containing another multiple-bidirectional leader recorded at a lower frame rate of 8000 fps. The multiple-bidirectional leader induced the 4th RS of Flash F triggered at 08:20:04 (UTC), 5 June 2019. The leader first entered the field of view in Frame 1 and then extinguished in Frame 2. A luminous segment whose tail was higher than the head of the just terminated leader occurred in Frame 3. Frames 1 through 3 may record the propagation of a stepped leader or the propagation of an attempted leader and a subsequent bidirectional attempted leader. The luminous segment will be referred to as the first BL below. The first BL extinguished in Frame 4 of which the speed of the upward positive end and the downward negative end were 4.66 × 10^4^ and 1.12 × 10^5^ m/s respectively. The second BL with quite low luminosity originated in Frame 5. The negative end of the second BL propagated downwards continuously and then induced the return stroke. From Frames 5 to 10, the positive end of the BL moved upward with an average speed of 9.29 × 10^4^ m/s, while the negative end extended downward with an average speed of 1.96 × 10^5^ m/s.

Figure 1h shows Case 8 mentioned in^23^ containing a BL that was caught up by another leader in Flash D. The chased BL and the chasing leader both propagated in the same path and occurred subsequently. The chased leader entered the field of view 1.9 ms earlier than the chasing leader. The length of the luminous channel of the chased leader decreased gradually from Frames 1 through 4, indicating it may become weaker and weaker until it approached the head of the chasing leader in the next few frames. From Frames 5 through 8, the tail of the chased leader extended backward with increased luminosity when the head of the chasing leader was approaching. The bidirectional chased leader then connected with the chasing leader, forming a larger leader that eventually induced the 8th RS. From Frames 5 to 8, the positive end of the BL moved upward with an average speed of 1.81 × 10^6^ m/s, while the negative end extended downward with an average speed of 9.91 × 10^5^ m/s.

### Speed characteristics of BL development

**Figure 2:**
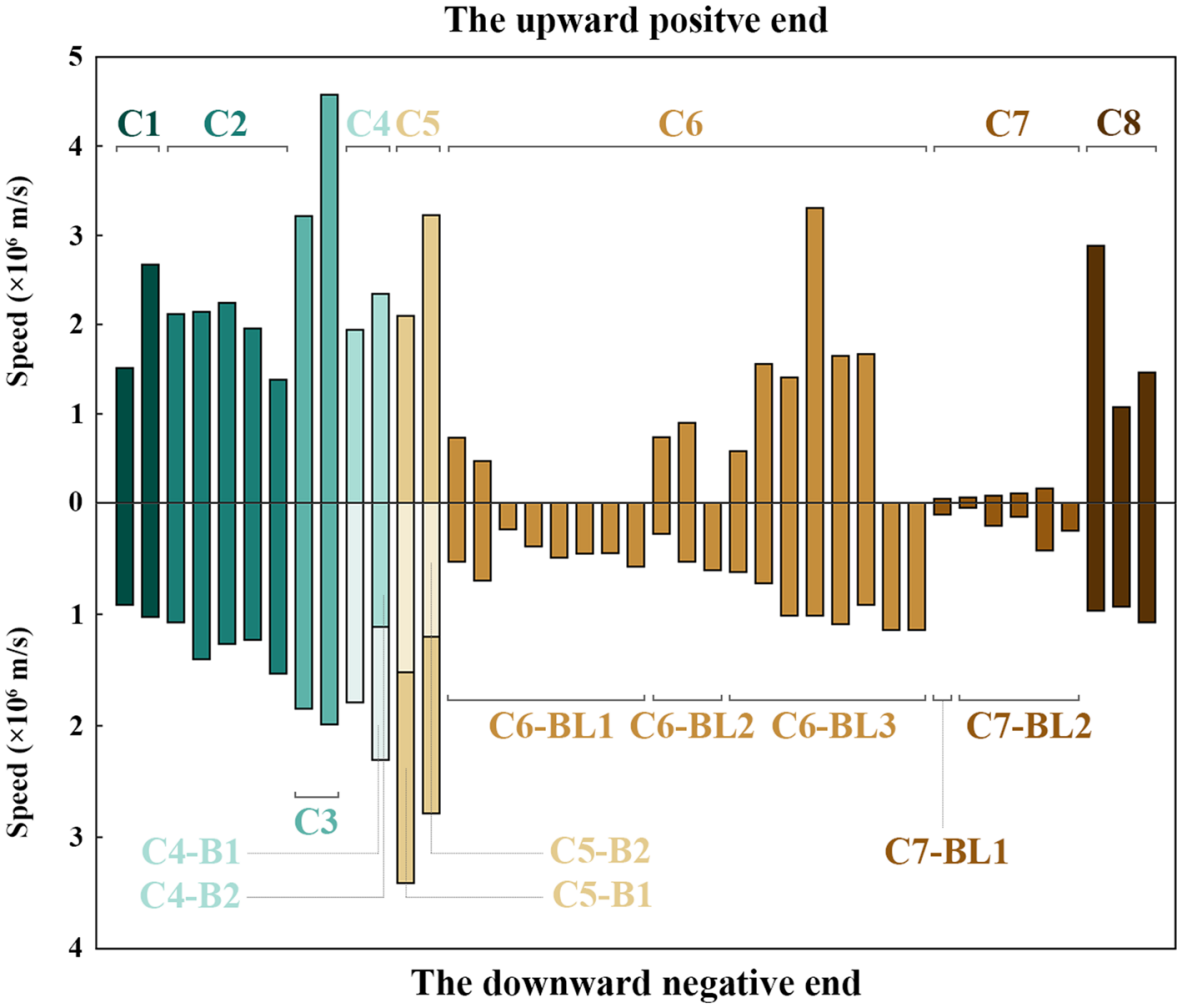
The propagation speed of two ends of BL development in eight cases. C1 represents Case 1, C2 represents Case 2, and so on. C4-B1, C4-B2, and C5-B1, C5-B2 represent the two branches of the downward negative end in Case 4 and Case 5. C6-BL1 to BL3 represent the three BL development in Case 6. C7-BL1 to BL2 represent the two BL development in Case 7.

The propagation speed of the upward positive end and the downward negative end was exhibited in Fig. 2 for each BL development in eight cases. The propagation speed of the upward positive end is absent in C6-BL1 to C6-BL3 and C7-BL2. That was because the upward positive end reached the top of view of the HSC as shown in Fig. 1. Except for Case 7, the upward positive end traveled faster than the downward one in the other seven cases. The lower speed of the upward positive end in Case 7 may be related to the zigzag propagating path and the calculation error caused by reaching the top of view of the HSC.

The observation results on the propagation speed of two ends of BL development were in accordance with the results observed by Qie et al.^4^. Qie et al.^4^ observed two rocket-triggered lightning flashes of which the positive leader propagated with an average speed of 1.3 × 10^6^ m/s and 2.2 × 10^6^ m/s, roughly twice as fast as its negative counterpart with the speed of 7.8 × 10^5^ m/s and 1.0 × 10^6^ m/s, respectively. In our observation, BLs usually initiated around the place where the previous downward dart or stepped leaders decayed. So, there were residual negative charges on the track of the previous downward leaders. When the BL development began the upward positive end went into the track. The neutralization reaction of positive and negative charges promoted the advance of the upward positive end. The downward negative end moved toward the ground along the discharge path of the previous return stroke. Compared with the upward positive end, the residually same polarity charges and the cooler discharge path were not conducive to the rapid propagation of the downward negative end.

### Electric field features of downward leader processes related to seven cases

**Figure 3:**
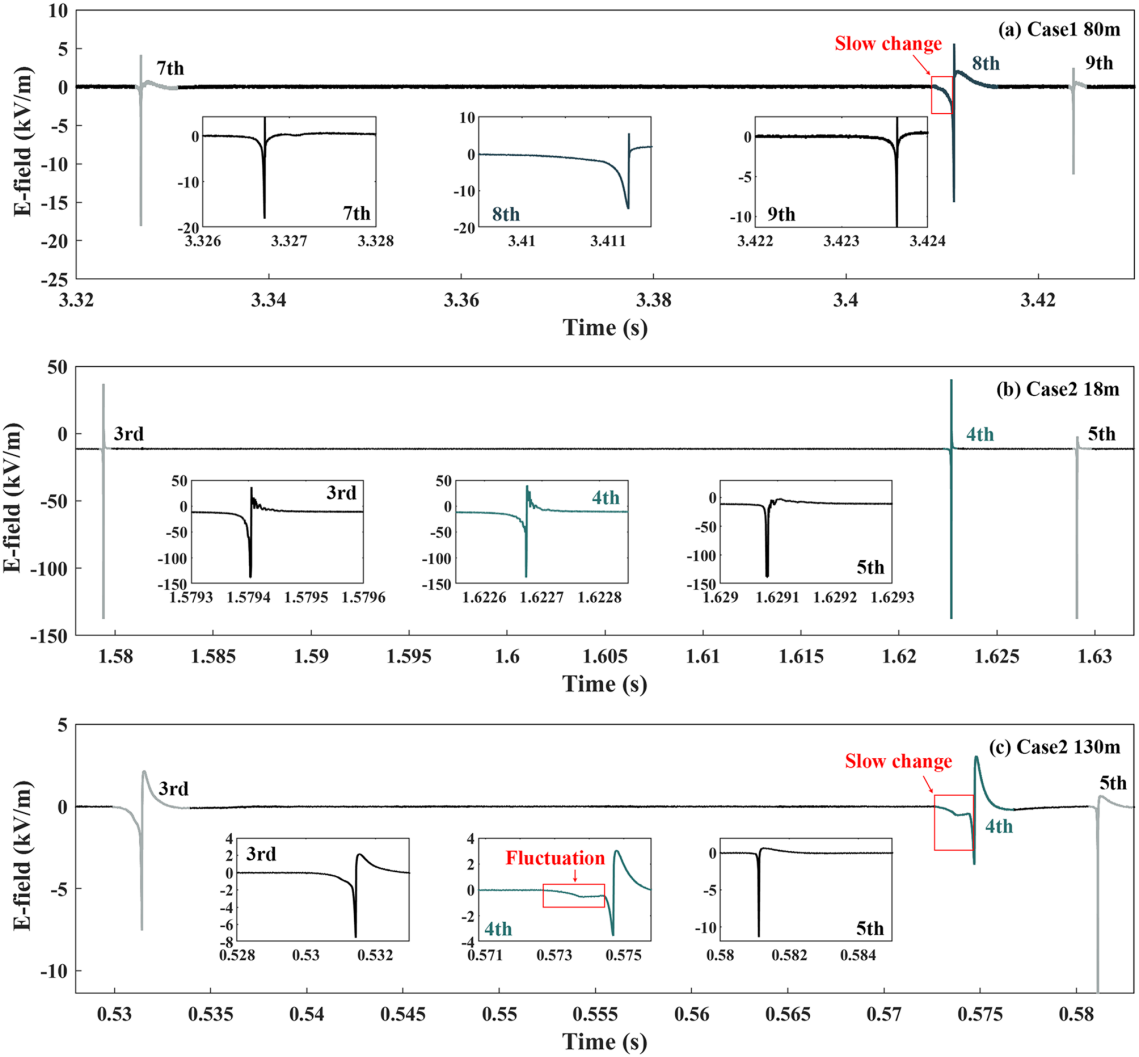
The E-field waveforms of Cases 1 and 2, along with their previous and subsequent downward leader–return stroke sequences.

**Figure 4:**
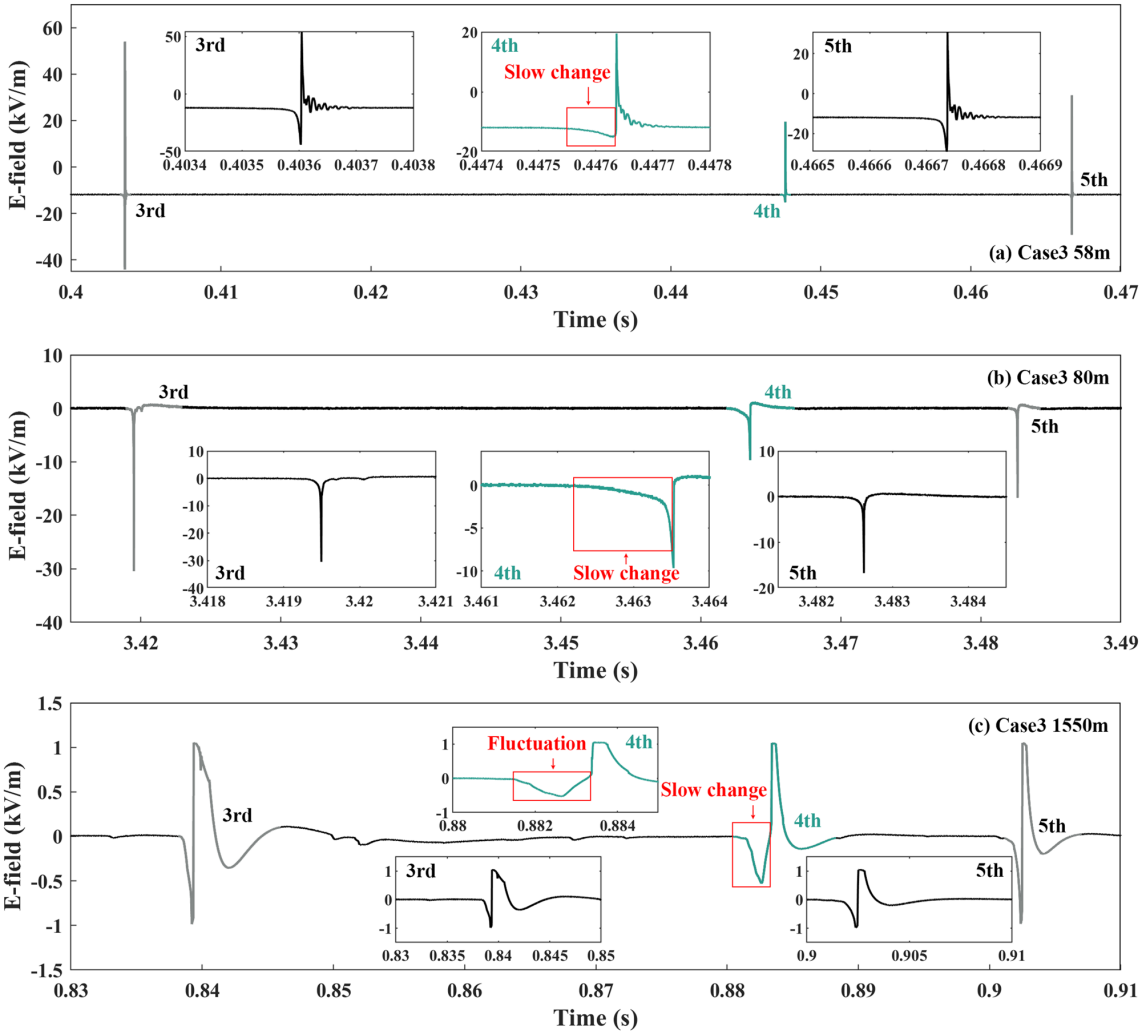
The E-field waveforms of Case 3 at different observation distances.

**Figure 5:**
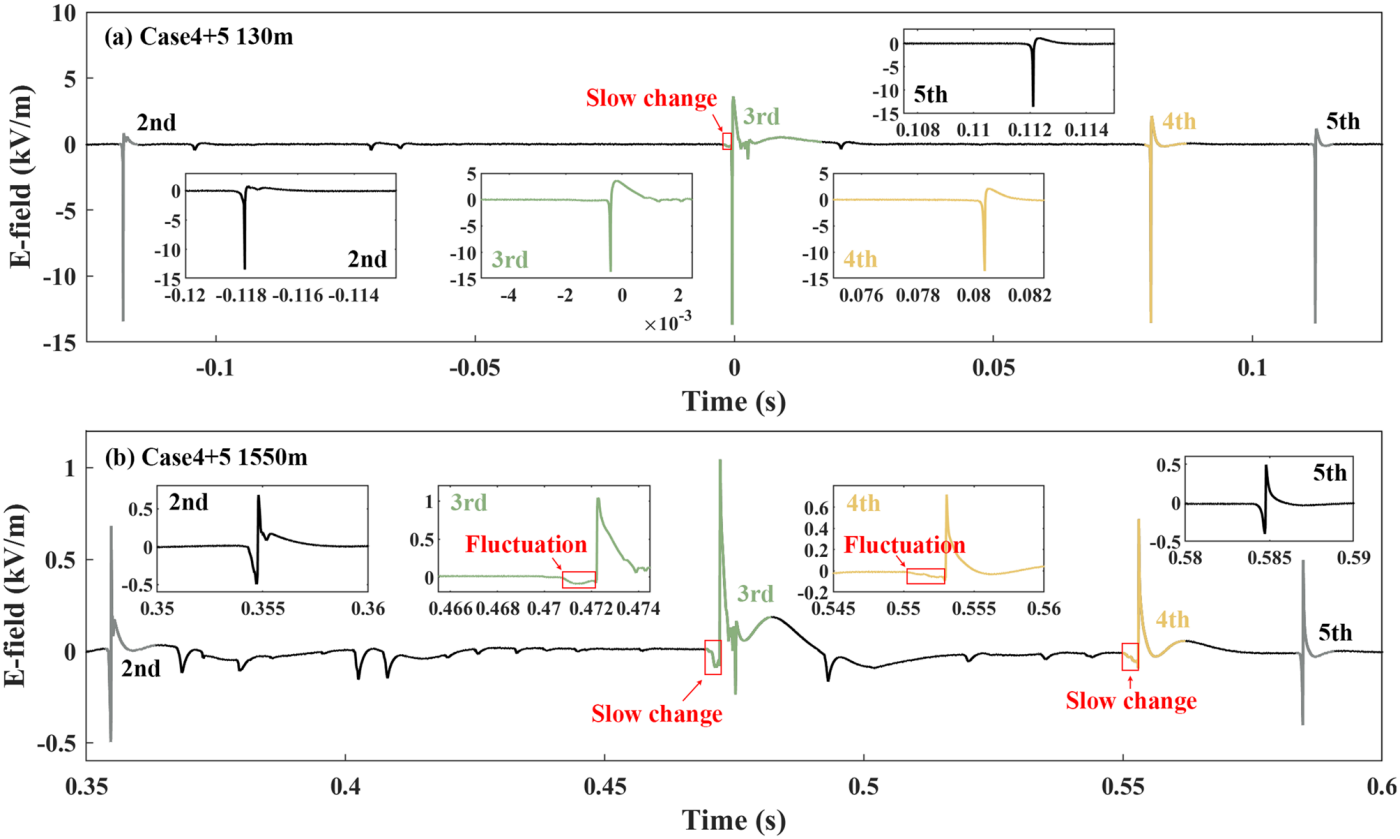
The E-field waveforms of Cases 4 and 5, along with their previous and subsequent downward leader–return stroke sequences.

**Figure 6:**
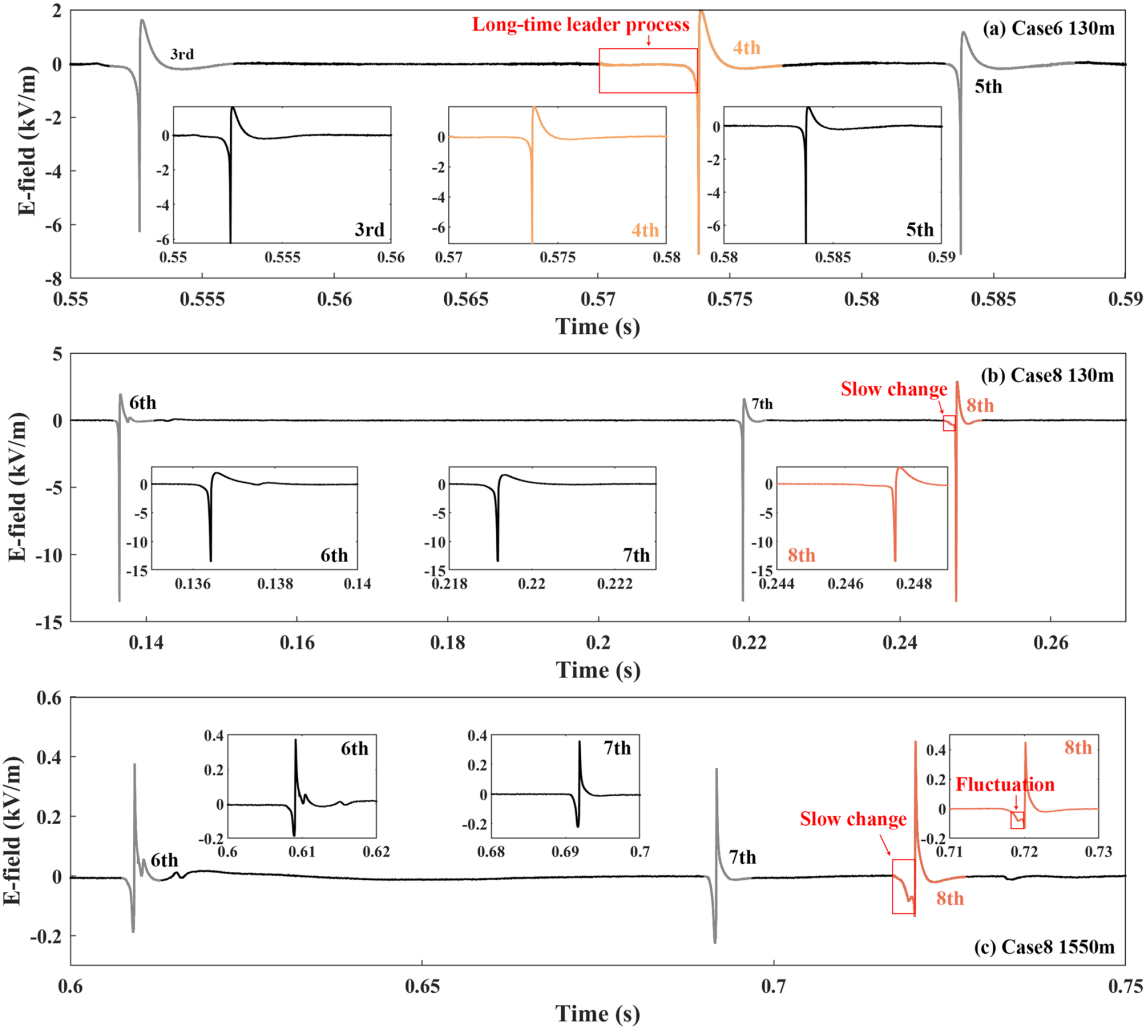
(**a**) The electric field waveform of Cases 6, along with its previous and subsequent downward leader–return stroke sequences. (**b-c**) The electric field waveforms of Case 8 and its previous two downward leader–return stroke sequences because the 8th RS was the last one in Flash D.

Figures 3, 4, 5 and 6 show the electric field (E-field) waveforms at different observation distances of downward leader processes related to seven cases. For comparison, the E-field waveforms of their previous and subsequent downward leader–return stroke sequences are also presented. In Fig. 3a there was a slow change during the downward leader process of the 8th RS (Case 1), compared with that of the 7th and 9th RSs in the E-field waveform at 80 m. The E-field waveform in Fig. 3c, compared with that in Fig. 3b, made the difference clearer between the downward leader processes of the 3rd, 4th, and 5th RSs. The slow change of the 4th RS was about twice as long as the fast decrease of the 5th RS, and its amplitude was about half of that of the fast decrease. The insert figure of the 4th RS (Case 2) in Fig. 3c exhibits not only a slow change but also a fluctuation during the downward leader process. The fluctuation referred to that the E-field waveform was not monotonically increasing in the negative direction preceding the RS. In Fig. 4, the E-field waveforms all presented slow changes before the 4th RS at 58 m, 80 m, and 1550 m. Especially, a fluctuation took place before the 4th RS (Case 3) in the E-field waveform at 1550 m.

Cases 4 and 5 were included in the downward leader processes of the 3rd and 4th RSs in Flash D, which was conducted as altitude-triggered lightning. Slow changes both appeared in the E-field waveforms at 130 m and 1550 m as shown in Fig. 5. There were fluctuations before the 3rd and 4th RSs in the E-field waveform at 1550 m.

In Fig. 6a, Case 6 presented a long-time downward leader process before the 4th RS in the E-field waveform at 130 m, which was caused by the multiple BL processes in Fig. 1f. The downward leader process before the 4th RS lasted for 1.85 ms in sight of the HSC while the downward leader processes before the 3rd and 5th RS lasted for 0.65 and 0.25 ms. Both the E-field waveforms at 130 m and 1550 m had slow changes before the 8th RS (Case 8) in Fig. 6b,c. A fluctuation was also in the E-field waveform at 1550 m before the 8th RS.

From Figs. 3, 4, 5 and 6 slow changes were recognized during downward leader processes in the E-field waveforms at 58 m, 80 m, 130 m, and 1550 m, not at 18 m. These slow changes made the downward leader processes of seven cases longer than those before the previous and subsequent downward leader–return stroke sequences. In addition, fluctuations occurred in the E-field waveforms at 130 m in some cases and the E-field waveforms at 1550 m in all cases.

### Electric field characteristics of the BL development of seven cases

The analysis in Part C shows that the downward leader processes of seven cases were changing slowly in the E-field at more than 80 m. In some cases, the E-field waveforms of the downward leader processes had non-monotonic fluctuations. In this part, we research the E-field features related to BL development.

**Figure 7:**
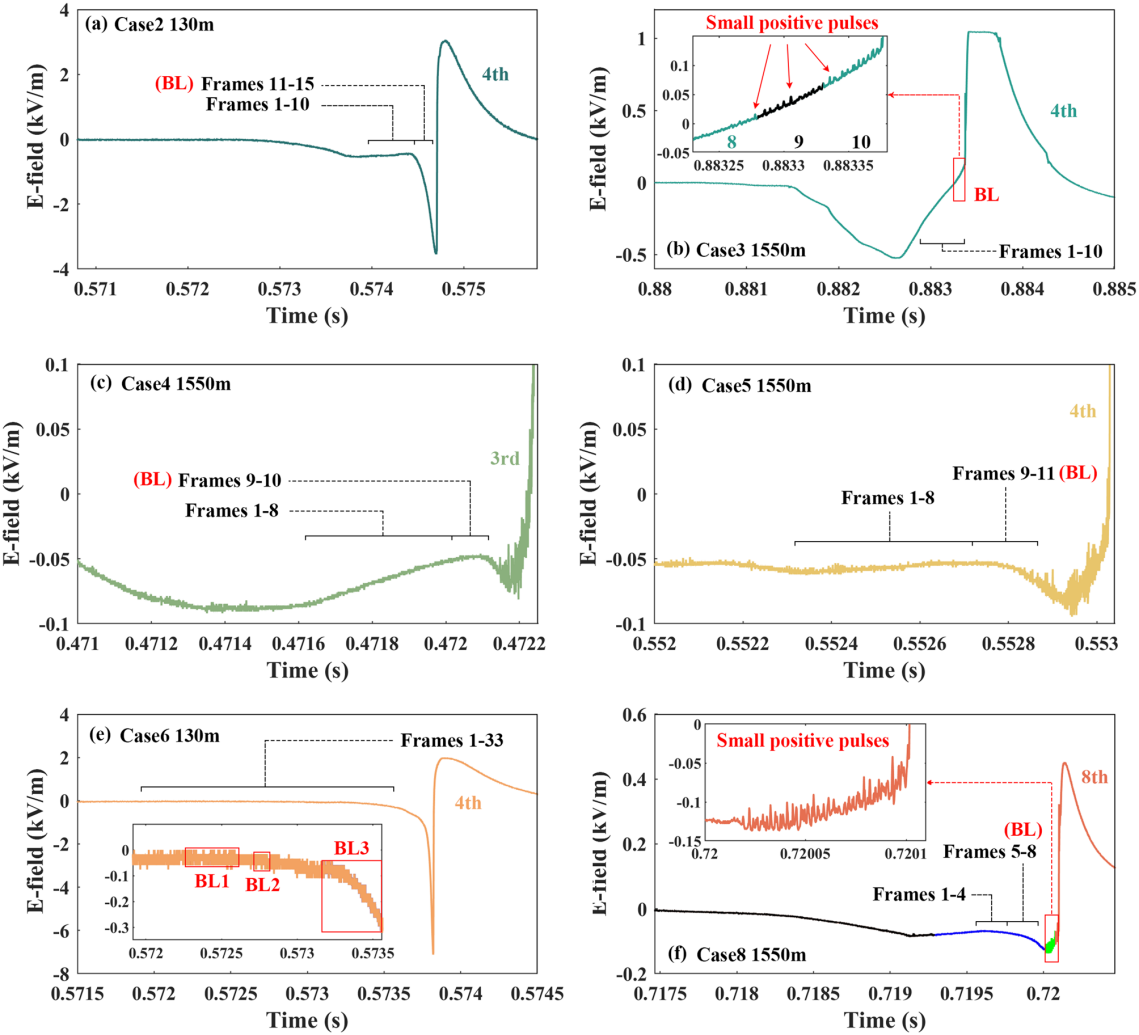
The E-field waveforms of the BL development of Cases 2–6 and 8.

In Fig. 7a, the BL development of Case 2 was contained in the fluctuation. The stagnation of the downward leader development led to the E-field no longer negatively increasing but entering a plateau period from Frames 1 to 10. Then the BL development from Frames 11 to 15 activated the negative change of the E-field waveform.

The E-field waveform of Case 3 was further enlarged in Fig. 7b. The waveform related to Frames 8 through 10 was colored with an uncertainty of 50 μs in the insert figure. The small positive pulses superimposed on the slowly increasing E-field waveform were scarce before the start of the bidirectional development or Frame 8. The unique slow decrease and the small positive pulses were the common features of Case 3 and the two cases reported by Qie et al.^4^. The BL development was accompanied by the positive increase of the E-field maybe because the 4th RS followed closely behind the BL development.

Cases 4 and 5 belonged to the two adjacent RSs of one Flash. The fluctuations of the E-field waveforms in Fig. 7c,d were related to the BL development of Cases 4 and 5. From Frames 1 to 8 in Fig. 7c,d, the E-field waveforms increased positively or entered a plateau period. When the BL development began the E-field waveforms changed negatively again. The difference between the two fluctuations in Fig. 7c,d was the positive increase in the amplitude of the E-field between the BL development. The state of the downward leader contributed to the difference. In Fig. 1d,e, the downward leader before the BL development of Case 4 had a higher dissipation than that of Case 5, resulting in a less negative influence on the E-field.

In Case 6, three BLs originated before the return stroke was induced in Fig. 7e. The intervals between the termination of a preceding attempted leader and the initiation of a BL were at least 149 μs, 99 μs, and 49 μs, respectively. Qie et al.^4^ reported the first two cases of BL in a preexisting channel in triggered lightning flashes. Only one BL originated in the two cases, and the intervals were about 160 μs and 300 μs, respectively.

In Fig. 7f, the waveform before the chasing leader entered the field of view of the high-speed camera was painted black, the waveform corresponding to the existence of the two leaders was painted blue, and the waveform after the connection of the chased leader and chasing leader was painted green. The BL development of Case 8 was included in the fluctuation of the E-field. From Frames 1 to 4 in Fig. 1h, the chased leader became faint gradually. After Frame 5 the BL development started, and the E-field changed negatively. The E-field waveform in the red box is enlarged in the small figure, in which a lot of small positive pulses were superimposed on the positive increase.

Cases 2, 4–5, and 8 show that the slow change and fluctuation of the downward leader E-field was related to the BL development. The dissipation of the downward leader slowed down the negative increase of the E-field. Once the BL development started, the downward negative end of the BL moved toward the ground. Meanwhile, the E-field negatively increased until the RS occurred. For Case 6 Multiple BL development made the downward leader process much longer without obvious fluctuation, compared with Case 2 at the same observation distance. The small positive pulses before the RSs could be seen in some cases, for example, Cases 3–5 and 8 at 1550 m. In Case 3 the BL development was close to the 4th RS by chance. Therefore, whether the small positive pulses are related to the BL development or the process just before the RS needs further study in the future.

## Discussion

We divided the eight cases of BL development reported here into three types based on their progression characteristics. The first type was a reflection type of BL development representing Cases 1 through 5. The second type was a discontinuity type with multiple-bidirectional leaders representing Case 6 and Case 7. The third type was an inducement type including the chased bidirectional leader and the chasing leader, representing Case 8 reported here.

**Figure 8:**
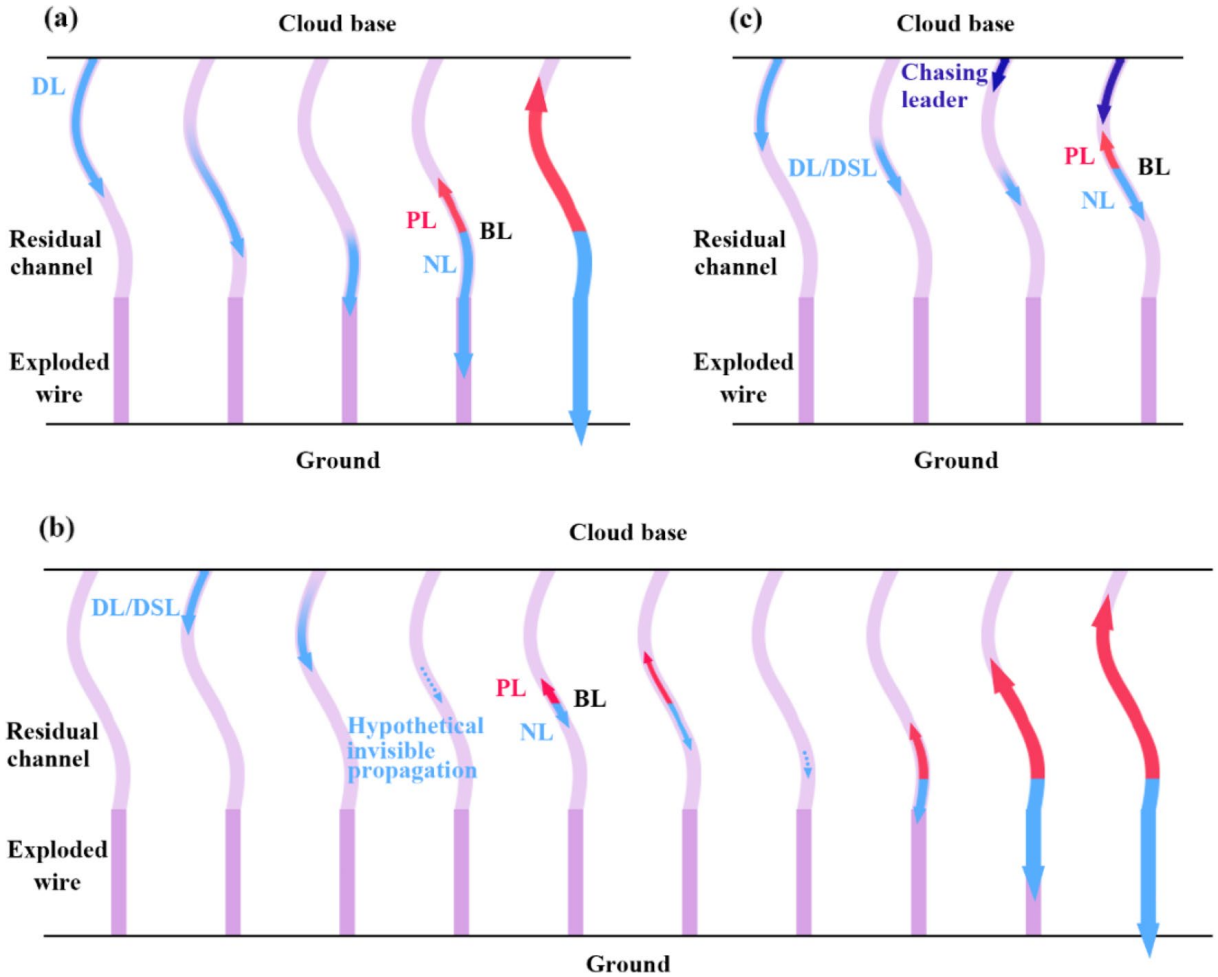
Concept maps of three types of bidirectional leader development. (**a**) A reflection type. (**b**) A discontinuity type. (**c**) An inducement type. *DL* dart leader, *PL* positive leader, *NL* negative leader, *BL* bidirectional leader, *DSL* dart-stepped leader.

Figure 8 shows a conceptual diagram of the three types of BL development in artificially-triggered lightning flashes. For the reflection type in Fig. 8a, the unidirectional leaders in Cases 1 through 3 turned to be the BLs when their leader heads reached the top of the exploded triggering wire, while the unidirectional leaders in Case 4 and Case 5 turned to be BLs when their leader heads reached the branch points. The top of the exploded triggering wire is the dividing point between the channel of the initial upward leader and the channel of the exploded triggering wire, where exists an impedance discontinuity. The branch points are the connection points between the main channel of a leader and the branches, where also exists an impedance discontinuity. The existence of the impedance discontinuity may result in the reflection of some portions of the leader current, causing the unidirectional leader to be bidirectional visually. Kotovsky et al.^24^ reported two attempted leaders that also had the visual reflection process.

In the discontinuity type, an initial dart leader or dart-stepped leader propagates downwards and then dies out or decays to be invisible. Then the first BL originates from a point below the head tip of the just terminated dart leader. The first BL may propagate continuously to reach the ground or die out soon to be an attempted leader. If the first BL dies out, the second BL may originate from a point below the head tip of the first BL. A similar process may cycle several times until a BL reaches the ground and induces the return stroke or all these leaders fail to reach the ground. It should note that the so-called termination here only means the channel of a leader become invisible or hard to be figured out in the high-speed speed frames. The two cases reported by Qie et al.^4^, which are the first two reported cases of BL in a preexisting channel in triggered lightning flashes, also belong to this type. Only one BL originated in the two cases reported by Qie et al.^4^, and the intervals were about 160 μs and 300 μs, respectively.

The inducement type of BLs develops from decaying unidirectional leaders. In the inducement type, the approach of the faster chasing leader is the key factor causing the unidirectional leader to be bidirectional. In this type, the chasing leader propagates downward along the residual discharge path with a faster speed compared with the chased leader. The gradually approaching chasing leader with negative head causes the decaying chased leader to polarize. The unidirectional chased leader turns to a bidirectional one. Different from the discontinuity type, the reflection type and inducement type of BLs were leaders propagating continuously without the invisible duration as recorded by the high-speed camera. At the same time, the occurrence of the reflection type and inducement type of BL is not the result of the initiation of a new leader but the result of the ends of a decaying unidirectional leader beginning to extend forward and backward.

The results in Part D present the features and differences of the E-field between the three types. For the reflection type (Cases 2, 4, and 5) and the inducement type, there are fluctuations of the downward leader E-field related to the BL development. The dissipation of the downward leader slows down the negative increase of the E-field. Once the BL development starts, the downward negative end of the BL moves towards the ground with the E-field negatively increasing. It should be pointed out here that if the BL development is closely followed by the RS (Case 3), the change of the E-field may be different. This situation needs further study. For the discontinuity type (Case 6), the close observation result shows no fluctuations in the E-field. The BL development has a much longer duration than the other two BL types.

## Methods

The eight cases of BL propagation were observed in an altitude-triggered and five classical-triggered lightning flashes in the summer of 2019 at the Guangzhou Field Experiment Site for Lightning Research and Testing in Conghua, Guangdong. Three electric field sensors (Sensors 1 to 3) and one electric field sensor (Sensor 4) were respectively installed at the launch site and the distant observation site. The distant observation site was 1.55 km away from the launch site. Sensors 1, 3, and 4 had the same equipment parameters, with a time constant of 1 ms and a 3 dB bandwidth ranging from 160 Hz to 500 kHz. Sensor 2 had a frequency bandwidth of ~ 80 Hz to 2 MHz and a decay time constant of 2 ms. At the launch site, there was a ground launcher and a tower launcher. The observation distances between Sensor 1 and the ground launcher and the tower launcher were respectively 58 m and 18 m. Sensor 2 was 80 m away from the ground launcher while Sensor 3 was 130 m away from the tower launcher. The electric field signal from Sensor 1 was recorded by an acquisition card having a sampling rate of 10 MS/s and a recording duration of 2 s. Three oscilloscopes were respectively used to record the electric field signals from Sensors 2, 3, and 4, with the recording duration of 5 s, 2 s, and 2 s and the sampling rate of 10 MS/s, 50 MS/s, and 5 MS/s. Oscilloscopes, the acquisition card, and the high-speed camera were synchronized through GPS. More details and the layout of other equipment could be found in these papers^25–27^.

Two types of high-speed cameras (HSCs) at the distant observation site, were adopted at different times^28^. The lightning flashes triggered before 30 June were recorded by a Phantom M310 HSC configured with a Nikon 16 mm lens, operating at a rate of 8,000 frames per second (fps). The exposure time of the M310 camera was 120 μs, so the dead time was 5 μs. The actual space size corresponding to each pixel taken by M310 was 1.94 × 1.94 m^2^. The Phantom V2512 HSC with the same 16 mm lens had been in use since 30 June, operating at a rate of 20,000 fps. The exposure time of the V2512 camera was 49 μs, so the dead time was 1 μs. The actual space size corresponding to each pixel taken by V2512 was 2.71 × 2.71 m^2^.

## Conclusion

In this paper, we reported eight cases of BL development in a residual channel of artificially-triggered lightning flashes through the records of the high-speed camera and electric field signature. We presented and described the BL development by the processed HSC images. We calculated the speed of the upward positive end and the downward negative end. The upward positive end usually moved faster than the downward negative end because of the residual negative charge in the channel. The RS with BL development exhibited slow changes in the downward leader process of the electric field, compared with the previous and subsequent RSs.

The eight cases of bidirectional leaders can be divided into three types: a reflection type, a discontinuity type, and an inducement type, for their unique progression characteristics. Reflection type and inducement type are first observed here, and the initiation of a bidirectional leader may occur more than once in the discontinuity type. In reflection type and inducement type, the bidirectional leaders develop from dart leaders whose luminous bodies are shorter and shorter. The tail of the dart leader begins to extend backward when the leader’s head reaches a dividing point in the reflection type or when the head of another leader is close enough in the inducement type. Two cases of discontinuity type are observed, and both are multiple-bidirectional leaders observed for the first time. For the reflection type and the inducement type, there are fluctuations of the downward leader E-field related to the BL development. Before the BL development, the dissipation of the downward leader slows down the negative increase of the E-field. Once the BL development starts, the downward negative end of the BL moves towards the ground with the E-field negatively increasing. For the discontinuity type, the close observation result shows no fluctuations in the E-field. The BL development has a much longer duration than the other two BL types. In some cases, small positive pulses appear simultaneously in the BL development and before the RS process.

## Data Availability

Correspondence and requests for data and materials should be addressed to J.G.W. or L.C.

## References

[1] Kasemir HW (1960). A contribution to the electrostatic theory of a lightning discharge. J. Geophys. Res..

[2] Tran MD, Rakov VA (2016). Initiation and propagation of cloud-to-ground lightning observed with a high-speed video camera. Sci. Rep.-UK.

[3] van der Velde OA, Montanya J (2013). Asymmetries in bidirectional leader development of lightning flashes. J. Geophys. Res.-Atmos..

[4] Qie X (2017). Bidirectional leader development in a preexisting channel as observed in rocket-triggered lightning flashes. J. Geophys. Res. Atmos..

[5] Gamerota WR (2014). Dart-stepped-leader step formation in triggered lightning. Geophys. Res. Lett..

[6] Mazur V (1989). A Physical model of lightning initiation on aircraft in thunderstorms. J. Geophys. Res.-Atmos..

[7] Shao XM, Krehbiel PR (1996). The spatial and temporal development of intracloud lightning. J. Geophys. Res. Atmos..

[8] Warner, T. A. *et al.* Characteristics of upward leaders from tall towers. in *22nd ILDC*, *Broomfield, Mar 2012* (2012).

[9] Castellani A (1998). Laboratory study of the bi-leader process from an electrically floating conductor. Part I: General results. IEEE Proc. Sci. Meas. Technol..

[10] Lalande P (1998). Leader properties determined with triggered lightning techniques. J. Geophys. Res.-Atmos..

[11] Lu WT (2009). Simultaneous optical and electrical observations on the initial processes of altitude-triggered negative lightning. Atmos. Res..

[12] Kostinskiy AY (2015). Observation of a new class of electric discharges within artificial clouds of charged water droplets and its implication for lightning initiation within thunderclouds. Geophys. Res. Lett..

[13] Montanya J, van der Velde O, Williams ER (2015). The start of lightning: Evidence of bidirectional lightning initiation. Sci. Rep.-UK.

[14] Warner TA, Saba MMF, Schumann C, Helsdon JH, Orville RE (2016). Observations of bidirectional lightning leader initiation and development near positive leader channels. J. Geophys. Res.-Atmos..

[15] Gorin, B. N., Levitov, V. I. & Shkilev, A. V. *Some Principles of Leader Discharge of Air Gaps with a Strong Non-Uniform Field*. 274–278 (1976).

[16] Mazur V, Ruhnke LH, Warner TA, Orville RE (2013). Recoil leader formation and development. J. Electrostat..

[17] Mazur V (2016). The physical concept of recoil leader formation. J. Electrostat..

[18] Saba MMF (2008). Positive leader characteristics from high-speed video observations. Geophys. Res. Lett..

[19] Montanyà J (2012). High-speed video of lightning and X-ray pulses during the 2009–2010 observation campaigns in northeastern Spain. Atmos. Res..

[20] Jiang R, Wu Z, Qie X, Wang D, Liu M (2014). High-speed video evidence of a dart leader with bidirectional development. Geophys. Res. Lett..

[21] Jiang R, Yuan S, Qie X, Liu M, Wang D (2022). Activation of abundant recoil leaders and their promotion effect on the negative-end breakdown in an intracloud lightning flash. Geophys. Res. Lett..

[22] Wang F (2021). Leader-chasing behavior in negative artificial triggered lightning flashes. Sci. Rep..

[23] Wang JG (2022). Observation of five types of leaders contained in a negative triggered lightning. Sci. Rep.-UK.

[24] Kotovsky DA, Uman MA, Wilkes RA, Jordan DM (2019). High-speed video and lightning mapping array observations of in-cloud lightning leaders and an M component to ground. J. Geophys. Res.-Atmos..

[25] Cai L (2022). Differences between triggered lightning striking to ground and distribution line inferred from measured currents and electromagnetic fields. High Volt.

[26] Cai L (2021). Optical progressing and electric field change characteristics of altitude—Triggered lightning flash with different development paths. J. Geophys. Res. Atmos..

[27] Cai L (2020). Characterization of magnetic field waveforms from triggered lightning attached on transmission line at 18 m, 130 m, and 1.55 km. High Volt.

[28] Wang J (2022). Two successive bidirectional leaders propagated in the triggered lightning channel. Sci. Rep..

